# Antimicrobial, Multidrug Resistance Reversal and Biofilm Formation Inhibitory Effect of *Origanum majorana* Extracts, Essential Oil and Monoterpenes

**DOI:** 10.3390/plants11111432

**Published:** 2022-05-27

**Authors:** Tasneem Sultan Abu Ghazal, Zsuzsanna Schelz, Lívia Vidács, Nikoletta Szemerédi, Katalin Veres, Gabriella Spengler, Judit Hohmann

**Affiliations:** 1 Department of Pharmacognosy, University of Szeged, H-6720 Szeged, Hungary; abu.ghazal.tasneem.sultan@stud.u-szeged.hu (T.S.A.G.); vidacs.livia@pharmacognosy.hu (L.V.); veres@pharmacognosy.hu (K.V.); 2 Department of Pharmacodynamics and Biopharmacy, University of Szeged, H-6720 Szeged, Hungary; schelz.zsuzsanna@szte.hu; 3 Department of Medical Microbiology, Albert Szent-Györgyi Health Center and Albert Szent-Györgyi Medical School, University of Szeged, Semmelweis utca 6, H-6725 Szeged, Hungary; szemeredi.nikoletta@med.u-szeged.hu (N.S.); spengler.gabriella@med.u-szeged.hu (G.S.); 4 Interdisciplinary Centre for Natural Products, University of Szeged, Eötvös u. 6, H-6720 Szeged, Hungary

**Keywords:** *Origanum majorana*, Lamiaceae, essential oil, antimicrobial effect, efflux pump inhibitor, inhibition of biofilm formation

## Abstract

*Origanum majorana* L. is a widely used medicinal plant; its distilled oil and preparations are extensively utilised in the phytotherapy and food industries. The objective of this study is to evaluate the extracts and the essential oil (EO) of *Origanum majorana* L, and its monoterpenes for antimicrobial, bacterial multidrug resistance reversing, and biofilm formation inhibitory potency. The composition of EO and *n*-hexane extract was characterized by GC-MS. In the essential oil terpinen-4-ol (24.92%), *trans*-sabinene hydrate (25.18%), γ-terpinene (6.48%), *cis*-sabinene hydrate (5.44%), *p*-cymene (4.72%), sabinene (4.53%), α-terpineol (4.43%), and α-terpinene (3.00%) were found as the main constituents while *trans*-sabinene hydrate (1.43%), and terpinen-4-ol (0.19%) were detected in the *n*-hexane extract besides a series of hydrocarbons. The antibacterial activity of EO and terpinen-4-ol, α-terpinene, and linalool was also assessed against sensitive and drug-resistant *S. aureus*, and *E. coli* strains with MIC values of 0.125–0.250% and 30–61 µM, respectively. In the efflux pump (EP) inhibitory assay, made by the ethidium bromide accumulation method in *E. coli* ATCC 25922, and AG100 and *S. aureus* ATCC 25923, and MRSA ATCC 43300 strains, EO exhibited substantial activity, especially in the *E. coli* ATCC 25922 strain. Among the EO constituents, only sabinene was an EP inhibitor in sensitive *Escherichia* strain. In the case of *S. aureus* strains, EO and sabinene hydrate exhibited moderate potency on the drug-resistant phenotype. The antibiofilm effects of the samples were tested by crystal violet staining at sub-MIC concentration. γ-Terpinene, terpinen-4-ol, sabinene, sabinene hydrate and linalool were found to be effective inhibitors of biofilm formation (inhibition 36–86%) on *E. coli* ATCC 25922 and *S. aureus* MRSA ATCC 43300, while EO was ineffective on these strains. In contrast to this, biofilms formed by *E. coli* AG100 and *S. aureus* ATCC 25923 were significantly inhibited by the EO; however, it was not affected by any of the monoterpenes. This observation suggests that the antibiofilm effect might be altered by the synergism between the components of the essential oil.

## 1. Introduction

*Origanum majorana* L. (syn. *Majorana hortensis* Moench, widely known as ‘sweet marjoram’) is a 30–50 cm tall herb in the Lamiaceae family. This herb has been widely utilized as a medicinal plant and for flavoring food since ancient times. *O. majorana* is native to the Mediterranean region; however, it was cultivated in many countries of Eastern Europe, North Africa, Middle Asia, and South and North America. Apart from extensively utilizing it as a condiment to flavour meats, salads, and soups, it was also found that this herb has a great potential for industrial applications as an antimicrobial and antioxidant ingredient. In the food industry, the distilled oil is frequently applied to increase storing stability and reduce microbial contamination [1,2]. The essential oil is also used in perfumery, pharmaceuticals and cosmetics. In Hungary, sweet marjoram is one of the oldest and most widely used herbs and it can be grown there in high quality.

In traditional medicine, the dried leaves, leaf extract, and essential oil of sweet marjoram were used for the treatment of respiratory, gastrointestinal, and urinary tract disorders, and as a spasmolytic, antirheumatic, diuretic, and antiasthmatic remedy [3]. Various pharmacological properties of *O. majorana* have been proven, including antioxidant, antibacterial, hepatoprotective, cardioprotective, antiulcer, anticoagulant, anti-inflammatory, antiproliferative, and antifungal activities. Several studies also demonstrated the antibacterial and antifungal activities of the EO and its constituent terpinen-4-ol against some Gram-positive and Gram-negative bacteria, and fungal strains, including some drug-resistant clinical isolates [4,5,6,7,8,9,10,11]. The combined effect of the essential oil of sweet marjoram and beta-lactam antibiotics against beta-lactamase-producing *Escherichia coli* was studied, but no interaction was revealed [12]. Furthermore, Kerekes et al. [13,14] investigated the effect of sweet marjoram and terpinen-4-ol on single- and dual-species biofilms, which can cause food quality problems and/or serious food-borne infections produced on food surfaces. In these studies, the antibiofilm formation preventive activity of sweet marjoram essential oil was confirmed against food-related microorganisms.

From a phytochemical point of view, *O. majorana* is a rich source of terpenoids. Monoterpene EO constituents, diterpenes and triterpenes are its characteristic compounds. In addition, the occurrence of flavonoid aglycons and glycosides, hydroxyquinone derivatives (hydroxyquinone, arbutin, methylarbutin), tannins, and phenolic acids (rosmarinic acid, caffeic acid, caftaric acid, chlorogenic acid, protocatechuic acid) were described from the hydroalcoholic, ethyl acetate, and water extracts of the plant [2,3]. Phytochemical studies on *O. majorana* essential oil (EO) have reported variations in the volatile compositions according to geographical origins. While monoterpenes have been reported to be the dominant constituents of EO and some monoterpenes are responsible of its antimicrobial activity (e.g., sabinene hydrate and terpinen-4-ol) in most of the studies, sesquiterpenes was mentioned as a lower content constituent. Other studies have evaluated the EO isolated from *O. majorana* and found that different chemotypes exist (e.g., sabinyl, terpineol, carvacrol, and mixed sabinyl/α-terpineol chemotypes) [15]. Tabanca et al. reported that *O. majorana* native to the southeastern Mediterranean region of Turkey can be characterized by a high carvacrol content in its EO, while the *O. majorana* growing in the western part of Turkey contains only trace amounts of carvacrol, and *cis*-sabinene hydrate (30–44%) and terpinen-4-ol (8–14%) were found as the main constituents. In contrast to this, European type *O. majorana* can be characterized by high amounts of terpinen-4-ol (>20%) and *cis*-sabinene hydrate (3–18%) [16].

The present study aims to provide a broader insight into the effects of sweet marjoram extracts and essential oil against human pathogen microorganisms. Methanolic extract, *n*-hexane extract and EO obtained by hydrodistillation were analyzed against nine bacterial and one fungal strains by disc diffusion and microdilution methods. The chemical composition of the *n*-hexane extract and EO was characterized by GC-MS and GC-FID measurements. For the first time, the six main compounds of the essential oil (terpinen-4-ol, sabinene, sabinene-hydrate, α-terpinene, γ-terpinene, and linalool) were also included in the microbiological examination in order to gain information about the compounds responsible for the observed activities.

Our work also aims to evaluate the bacterial multidrug resistance (MDR) reversal activity of the extracts, EO, and its compounds. MDR can reduce the efficacy of antibacterial therapy and since an effective treatment should be administered to the patient, MDR is an alarming problem to be resolved. For this reason, new antibacterial agents that interfere with several virulence factors (e.g., efflux pumps, biofilm formation) contributing to MDR could provide an alternative to overcome MDR in bacteria [17]. The bacterial MDR-modifying activity was investigated by real-time ethidium bromide accumulation assay on Gram-negative and Gram-positive bacteria. 

In addition, the extracts and monoterpene compounds were investigated for biofilm formation inhibitory activity. Biofilms are structured communities of microorganisms that live in a self-produced extracellular matrix and are formed to promote bacterial survival in hostile environments. Biofilms pose a serious problem for antimicrobial therapy because of the increased resistance of biofilm-associated organisms to antibiotics.

## 2. Results

### 2.1. Determination of the Composition of Essential Oil and n-Hexane Extract 

**Figure 1:**
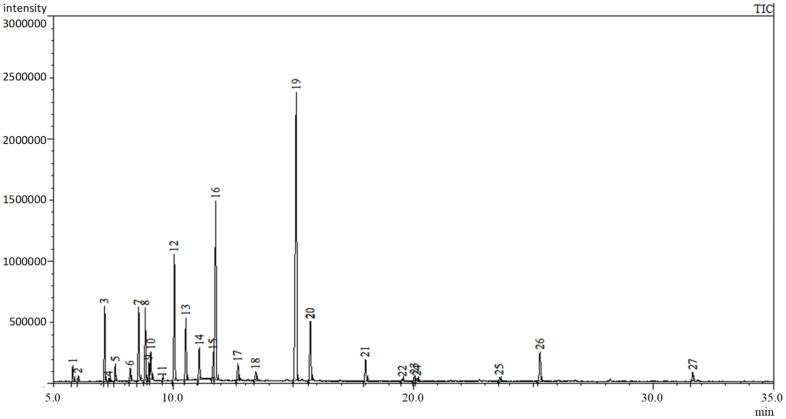
Total ion chromatogram of the essential oil of *Origanum majorana*.

The hydrodistillation of the aerial parts of the Hungarian variety of *O. majorana* afforded a pale-yellow oil with a 1.04% yield (*v/w*%). Table 1 and Figure 1 show the composition of the essential oil determined by GC and GC-MS; compounds are listed in Table 1 according to their elution on the column. Twenty-seven compounds were identified, accounting for 94.67% of the total oil. In the essential oil of marjoram, monoterpenes predominated, terpinen-4-ol (24.92%), *trans*-sabinene hydrate (25.18%), γ-terpinene (6.48%), *cis*-sabinene hydrate (5.44%), *p*-cymene (4.72%), sabinene (4.53%), α-terpineol (4.43%), and α-terpinene (3.00%) were found as the main constituents. Sesquiterpenes (β-caryophyllene and spathulenol) constitute only 2.45% of the EO. The composition of the *n*-hexane extract was also analyzed by GC-MS, and a much lower concentration of the EO constituents could be determined; its *trans*-sabinene hydrate content was found to be 1.43%, while terpinen-4-ol concentration was 0.19%. Moreover, a series of hydrocarbons could be detected in this extract (Table S1, Figure S1).

### 2.2. Antibacterial Assay

The antimicrobial activity of MeOH, n-hexane extracts, and the essential oil of O. majorana was tested against nine microorganisms, both Gram-positive (4), Gram-negative (4) and fungal strains (1). The results are summarized in Table 2 (inhibition zones in the agar diffusion assay) and Table 3 (MIC values). The MeOH extract was inactive or had only marginal activity against bacterial and fungal strains in the tested concentration, but the distilled essential oil (EO) exerted a remarkable antibacterial effect (10–14 mm) on the tested strains when applied in undiluted form (with the exception of Enterococcus faecalis and Pseudomonas aeruginosa where only marginal activity, 7, 8 mm, was detected). Comparing the antibacterial activity of the n-hexane extract and EO in the same diluted form (50 mg/mL), the *n*-hexane extract displayed a somewhat better effect than EO in contrast to its lower terpinen-4-ol and trans-sabinene hydrate content, suggesting the presence of other antibacterial compounds. The antibacterial effect of EO could be observed only in 20-fold higher concentration in undiluted form.

The EO possessed MIC values of 0.125% when tested against *S. aureus* ATCC 25923, *S. aureus* MRSA ATCC 43300, and *E. coli* ATCC 25922 strains, only *E. coli* AG100 seemed less susceptible (MIC 0.250%). This phenomenon raises the possibility that EO components might be the substrate of multidrug efflux pumps since *E. coli* AG100 bacterial strain is carrying the energy-dependent transporter AcrAB-TolC. The main constituents of the essential oil were also evaluated for antimicrobial activity against those microorganisms that were susceptible in the case of EO. It was found that α-terpinene and terpinen-4-ol were effective against *S. aureus* strains with MIC values of 61 and 60 mM, respectively, while linalool, α-terpinene and terpinen-4-ol showed antibacterial effect against *E. coli* strains with MIC values in the range of 30–61 mM (Table 4). The highest potency was exhibited by terpinen-4-ol against *E. coli* strains (MIC = 30 mM).

### 2.3. Real-Time Ethidium Bromide Accumulation Assay

In this study, the extracts, EO and its compounds were investigated for their ability to inhibit efflux pumps on Gram-negative (*E. coli*) and Gram-positive (*S. aureus*) model bacterial strains. The activity on efflux pump inhibition was assessed by real-time fluorimetry applying a fluorochrome (e.g., ethidium bromide) that is a substrate of the bacterial efflux pumps. In the real-time ethidium bromide (EB) accumulation assay, compounds with an efflux pump inhibitory (EPI) effect have a higher relative fluorescence index (RFI) compared to the untreated control.

The MeOH extract of sweet marjoram did not modify the EB accumulation in the tested 0.0312–0.125% concentration (Table 5). The n-hexane extract showed an effect on intracellular EB accumulation (RFI 0.39) only in the S. aureus MRSA ATCC 43300 strain. The EO of *O. majorana* revealed promising effects on EB accumulation. Comparing the positive controls CCCP and RES, EO exerted a pronounced inhibitory effect on efflux mechanisms by resulting RFIs at 50 and 100 μM in the same magnitude as the positive control substances in *E. coli* ATCC 25922 and *E. coli* AG100. This inhibitory effect could be detected both on drug resistant and sensitive *E. coli* strains. Among the EO constituents, sabinene had a notable inhibitory effect on *E. coli* ATCC 25922. On *S. aureus* MRSA ATCC 43300 EO and sabinene hydrate, contained in both the effective n-hexane extract and the essential oil, exhibited moderate inhibitory effects with RFI values of 0.35 and 0.27, respectively. However, the two samples did not show remarkable inhibition on the methicillin-susceptible Staphylococcus strain. One possible explanation for the higher effectiveness in the resistant strain is that the tested samples might inhibit the bacterial efflux pump systems resulting in the accumulation of themselves and other xenobiotic or antibiotics [18]. The n-hexane extract increased the EB accumulation (RFI 0.39) of *S. aureus* MRSA ATCC 43300 in a similar way to the EO, which indicates the presence of some active non-volatile compounds.

### 2.4. Biofilm Formation Inhibitory Effect

The effect of the extracts, EO and its monoterpene constituents on the biofilm formation of sensitive and resistant *S. aureus* and *E. coli* strains was evaluated. The extracts in 0.0312–0.125% and pure compounds in 50 and 100 µM concentrations were tested for their ability to decrease the formation of biofilm.

The values for the inhibition of biofilm formation are given in percentages in Table 6 and Table 7, based on the DMSO treated negative control values, which are considered to provide the lowest inhibition in the experimental set-up. Values over 30% were considered significantly high inhibitory effects [19]. *n*-Hexane extract, EO, γ-terpinene, and sabinene, sabinene-hydrate inhibited the biofilm formation of *E. coli* ATCC 25922, while MeOH extract, EO, terpinen-4-ol, sabinene, and sabinene-hydrate showed inhibitory effects in *S. aureus* MRSA ATCC 43300. Sabinene and sabinene-hydrate seemed to be the most potent of the investigated compounds, since these two monoterpenes exerted pronounced effects independently from cell wall structure of the bacteria. The inhibition was even more pronounced in lower concentrations (50 μM). *E. coli* AG100 and *S. aureus* ATCC 25923 form only weak biofilm, which could explain the lack of antibiofilm effects.

## 3. Discussion

Our investigations demonstrated the antibacterial, efflux pump inhibitory, and antibiofilm-forming effect of sweet marjoram EO and its main monoterpenes against the tested bacteria.

The antibacterial activity of the EO was shown against sensitive and drug resistant *S. aureus*, and *E. coli* strains with MIC values of 0.125–0.250% (Table 3). These data are in accordance with previous studies which proved the inhibitory activity of sweet marjoram EO against *S. aureus* and *S. pyogenes* with MICs of 125 and 250 µg/mL, respectively [20]. The *n*-hexane extract containing the same compounds as EO in a lower concentration revealed only weak activity in the disc diffusion test (7–8 mm). In our assay, the MeOH extract was almost inactive against bacterial strains, however, previously some papers were published on the antimicrobial activity of MeOH or EtOH extract of *O. majorana.* In these studies, higher concentrations and/or other bacterial strains were applied (MIC value 4 mg/mL against *S. aureus* clinical isolate, and *S. aureus* ATCC 29213) [10].

Among the tested six main monoterpenes of EO (terpinen-4-ol, sabinene, sabinene-hydrate, α-terpinene, γ-terpinene, and linalool), mainly terpinen-4-ol, α-terpinene, and linalool contribute to the antibacterial activity of EO, as demonstrated by their MIC values between 30–61 mM (Table 4). Our data are in agreement with earlier reports on the antimicrobial effect of these compounds [21,22]. Differences could not be observed between the pairs of sensitive and resistant strains. Among the EO constituents terpinen-4-ol can be highlighted because of its highest concentration in EO, and highest activities against *E. coli* ATCC 25922 and *E. coli* AG100 (30 mM). 

The emergence and ongoing spread of multidrug-resistant (MDR) bacteria is a major global public health problem. The inappropriate, overuse of antibiotics in the treatment of microbial infections is held to be the major reason contributing to the appearance of strains with reduced susceptibility to antibiotics. With the aim of re-sensitizing antibiotic-resistant bacteria, efflux pump inhibition could be applied to overcome the resistant phenotype [23,24]. Bacterial pathogens well known for their drug resistance are *S. aureus* and *E. coli*, which cause a very high global mortality rate among the humans infected. 

In our work, extracts of EO of *O. majorana* and its monoterpenes were assayed for efflux pump inhibitory activity in drug resistant and sensitive *S. aureus* and *E. coli* strains (Table 5). As mentioned previously, EO significantly increased the EB accumulation in all bacteria to some extent, especially in *E. coli* ATCC 25922. Among the EO constituents, only sabinene was an effective efflux pump inhibitor in the sensitive *E. coli* strain. In the case of *S. aureus* strains, EO and sabinene hydrate exhibited moderate potency on the drug-resistant phenotype. Therefore, Gram-negative, and Gram-positive bacteria have different susceptivity to efflux pump inhibitors, which can be explained by their different cell wall compositions [17].

Biofilm formation is a cyclical and dynamic process that includes different steps, starting from a reversible binding of planktonic cells to the surface followed by an irreversible attachment with the production of exopolysaccharides (EPS) that include proteins, lipids, polysaccharides, and DNA [25]. This primary biofilm structure matures over time and provides mechanical and chemical stability to the cells. EOs have a great potential in inhibiting biofilm formation. These lipophilic oily materials contain special plant metabolites in a concentrated form that often exert antimicrobial activity. The antibiofilm effect of EOs is well documented; they can interfere with different mechanisms involved in biofilm formation [19,26]. *E. coli* and *S. aureus* are among the most resistant biofilm-forming pathogenic bacteria. These bacteria occur ubiquitously, and they represent one of the most common causes of hospital- and community-acquired infections.

In our experiments, the antibiofilm effects of the extracts, EO and its monoterpenes were tested in sub-MIC concentration (MIC/2 or lower), and on resistant and sensitive *E. coli* and *S. aureus* strains were observed some activities (Table 6 and Table 7). Surprisingly, on *E. coli* ATCC 25922 and *S. aureus* MRSA ATCC 43300, the essential oil components γ-terpinene, terpinen-4-ol, sabinene, sabinene hydrate and linalool were effective biofilm formation inhibitors (inhibition 36–86%), while the essential oil was ineffective on these strains. In accordance with our findings, Kerekes et al. reported that *O. majorana* EO did not have inhibitory effect on biofilm formation, but their main components significantly inhibited the process in the case of Gram-positive bacteria (*B. cereus*) [13]. The Gram-negative bacterial biofilms (*E. coli* 0582) were inhibited by the EO, but the components were more effective. These results showed that the individual susceptibility of microbes is very different and plays a crucial role in the effectiveness of EOs and EO components [13]. The main target of these components is the cell wall and cytoplasmic membrane/or proteins embedded in the membrane.

Another interesting observation of our experiment was that biofilms formed by *E. coli* AG100 and *S. aureus* ATCC25923 were significantly inhibited by the sweet marjoram EO but it was not affected by any monoterpenes. Such a phenomenon has been described in the literature; several studies demonstrated a greater antibiofilm effect of EOs with respect to their main components, suggesting that minor constituents may have a synergistic effect with major bioactive compounds [26]. The antibiofilm formation effect of our samples was tested in two concentrations, but no clear concentration dependence could be noted.

In biofilm formation assay on *E. coli* ATCC 25922 and *S. aureus* MRSA ATCC 43300, substantial inhibitory activity of the MeOH extract was observed (Table 6 and Table 7). In the MeOH extract, the presence of phenolic acids, flavonoids, tannins, and triterpenes are expected and volatile compounds are in low concentration. The antibiofilm activity of the MeOH extract most probably can be explained by a high concentration of phenolic acids, rosmarinic acid, and chlorogenic acid [27]. Rosmarinic acid was reported to reduce biofilm formation of *S. aureus* in a concentration- and time-dependent manner in early-stage development [28]. RA also exerts inhibitory effects against *E. coli* K-12 and S. carnosus LTH1502 growth, through decreasing cell counts and cell numbers. The antibiofilm activity of chlorogenic acid against the clinical isolates of *Stenotrophomonas maltophilia* was displayed in vitro [29]. Other phenolic compounds, such as flavonoids, gallic acid, catechin may also have a contribution to antibiofilm activity [30].

## 4. Materials and Methods

### 4.1. Plant Material

Hungarian variety of *O. majoranae* was gathered at flowering stage from the production area in Bátya, Hungary in July 2020. The harvested plant was dried at room temperature (24–25 °C). The grower was Ferenc Okvátovity, the plant identification was made by K. Veres, Department of Pharmacognosy, University of Szeged. A voucher specimen (No. 896) was deposited in the herbarium of the Department of Pharmacognosy, University of Szeged. 

### 4.2. Solvents and Chemicals

Methanol and *n*-hexane used for extraction of the plant material were of analytical grade purity (Molar Chemicals Kft.). α-Terpinene (purity by GC > 95.0%), γ-terpinene (purity by GC > 96.5%), sabinene hydrate (purity by GC area ≥ 97.0%), linalool (purity by GC ≥ 99.0%), and (+)-terpinen-4-ol (purity by GC ≥ 98.5%, sum of enantiomers, enantiomeric ratio ~2:1) were purchased from Sigma-Aldrich (St. Louis, MO, USA), and sabinene from PhytoLab (Vestenbergsgreuth, Germany), purity by GC ≥ 75.0%.

### 4.3. Preparation of the Extracts for the Bioassays

A sample of 30 g of dried leaves of Hungarian variety of *O. majoranae* was extracted with 850 mL MeOH at room temperature (24 °C) in an ultrasonic bath. The methanolic extract was evaporated, a small portion (20 mg) used for antimicrobial assays, and the other main part (8.0 g) dissolved in 500 mL MeOH-H_2_O (1:1) and subjected to solvent-solvent partition with 5 × 150 mL *n*-hexane. This separation afforded 1.1723 g *n*-hexane extract (14.6%). 

### 4.4. Essential Oil Distillation

Three × 50 g dried and ground leaves samples of Hungarian variety of *O. majorana* were subjected to steam distillation for 3 h using a Clevenger-type apparatus. The yield of the pale-yellow essential oil was 1.04% (*v*/*w*) calculated to absolute dry leaves. Distillate of essential oil was dried over anhydrous sodium sulphate (Merck, Darmstadt, Germany), filtered (Whatman filter paper Grade 1), and stored at –20 °C until analyzed. The EO had penetrative aromatic odor.

### 4.5. Determination of the Composition of Essential Oil by GC and GC-MS 

Gas chromatography: The GC analysis was carried out with an HP 5890 Series II gas chromatograph (FID), using a 30 m × 0.35 mm × 0.25 µm HP-5 fused silica capillary column. The temperature program was from 60 °C to 210 °C at 3 °C min^−1^, and from 210 to 250 °C (2 min hold) at 5 °C min^−1^. The detector and injector temperature were 250 °C and the carrier gas was N_2_, with split sample introduction. The quantity of the individual components of essential oil was expressed as the percent of peak area relative to the total peak area from the GC/FID analysis.

Gas chromatography–mass spectrometry: GC–MS analysis was performed with a GC-MS-QP2010SE (Shimadzu) system. The temperature program was as above. The carrier gas was He at a linear velocity of 31.9 cm s^−1^ and the capillary column was ZB-5MS (30 m × 0.25 mm × 0.25 µm). The positive ion electron ionization mode was used, with ionization energy of 70 eV. Ion source temperature of 200 °C, interface temperature of 250 °C was applied, scan range was 40–550 m/z.

Identification of the compounds was based on comparisons with published MS data [31] and a computer library search (FFNSC) and by comparison of their retention indices with literature values [32]. Retention indices were calculated [33] against C8 to C32 *n*-alkanes on a ZB-5MS column. A mixture of aliphatic hydrocarbons was injected in hexane (Sigma–Aldrich, St. Louis, MO, USA) by using the same temperature program that was used for analyzing the essential oil.

### 4.6. Bacterial and Fungal Strains and Culture Conditions for Antimicrobial Assay

The test microorganisms used in this study were standard Gram-positive strains: *Staphylococcus aureus* (ATCC 29213), *Staphylococcus aureus* (MRSA) (ATCC 43300), *Staphylococcus epidermidis* (ATCC 12228), *Bacillus subtilis* (ATCC 6633), *Enterococcus faecalis* (ATCC 29212). The standard Gram-negative strains were *Escherichia coli* (ATCC 35218), *Escherichia coli* (AG-100), *Klebsiella pneumoniae* (ATCC 700603), and *Pseudomonas aeruginosa* (ATCC 27853). The fungal strain used was *Candida albicans* (ATCC 10231). Bacterial cultures were grown on standard Mueller–Hinton agar (MH) and fungal culture on RPMI plates (Diagnosticum Zrt.) at 37 °C under an aerobic environment overnight.

### 4.7. Determination of Antibacterial Activity by Disc Diffusion Method

Screening of antibacterial activity of compounds against standard bacterial and fungal strains for their inhibition zones was carried out by standard disc diffusion method. Concisely, the extracts and EO were dissolved in DMSO at the concentration of 50 mg/mL. Essential oil was also applied in undiluted form. The bacterial suspension (inoculums 0.5 McFarland, 1.5 × 10^8^ CFU mL^−1^) was spread on plate and the sterile filter paper discs [6 mm in diameter, Whatman antibiotic paper disc (Cytiva)] impregnated with 10 μL of the sample solution was placed. DMSO served as negative control, and discs containing antibiotics and antifungals were used as positive controls. The plates were incubated (37 +/− 2 °C for 20 h) under aerobic conditions. The diameters of inhibition zones produced by the compounds (including the disc) were measured and recorded in triplicate. An average zone of inhibition was calculated for the three replicates.

### 4.8. Determination of MIC Values

Minimum inhibitory concentration (MIC) of the samples was determined with the microdilution method, in a 96-well plate, according to the Clinical and Laboratory Standard Institute (CLSI) guidelines. The media used was MHB. The concentrations of pure compounds tested ranged from 100 mM to 0.195 mM, and extracts and essential oils from 10 µL/mL to 0.0195 µL/mL The MIC was determined by visual assessment. DMSO was used as a solvent in subinhibitory concentration (1% *v/v*). The values are given as the mean determined for three replicates from three independent experiments.

### 4.9. Bacterial Strains for Efflux Pump Inhibitory Assay

As Gram-negative strain, the wild-type *Escherichia coli* K-12 AG100 [argE3 thi-1 rpsL xyl mtl Δ(gal-uvrB) supE44], expressing the AcrAB-TolC efflux pump (EP) at its basal level and *E. coli* (ATCC 25922) strains were used. As Gram-positive strains, *Staphylococcus aureus* American Type Culture Collection (ATCC 25923) strain was used as methicillin-susceptible reference and the methicillin and oxacillin-resistant *S. aureus* MRSA ATCC 43300 strains were investigated in the study. 

### 4.10. Real-Time Ethidium Bromide Accumulation Assay

The effect of extracts and compounds on ethidium bromide (EB) accumulation was established by the automated EB method using a CLARIOstar Plus plate reader (BMG Labtech, UK). Tthe bacterial strain was first incubated until it reached an optical density (OD) of 0.6 at 600 nm. The culture was washed with phosphate buffered saline (PBS; pH 7.4) and centrifuged at 13.000× *g* for 3 min, the cell pellet was re-suspended in PBS. The pure compounds were added at 50 and 100 µM and the extracts and EO at MIC/2 concentration to PBS containing a non-toxic concentration of EB (2 µg/mL). Then, 50 μL of the EB solution containing the sample to be tested were transferred into a 96-well black microtiter plate (Greiner Bio-One Hungary Kft, Mosonmagyaróvár, Hungary), and 50 μL of bacterial suspension (OD_600_ 0.6) were put into each well. Reserpine (RES) was applied at 25 µM as a positive control on *S. aureus* strains, carbonyl cyanide 3-chlorophenylhydrazone (CCCP) was applied at 50 µM as a positive control on *E. coli* strains. Then, the plates were evaluated by the CLARIOstar plate reader, the fluorescence was measured at excitation and emission wavelengths of 525 and 615 nm every minute for one hour on a real-time basis. All experiments were performed in triplicate. From the real-time data, the activity of the samples, namely the relative fluorescence index (RFI) of the last time point (minute 60) of the EB accumulation assay, was calculated according to the following equation:

RFI = (RF_treated_ − RF_untreated_)/RF_untreated_

where RF_treated_ is the relative fluorescence (RF) at the last time point of EB retention curve in the presence of an inhibitor, and RF_untreated_ is the RF at the last time point of the EB retention curve of the untreated control with the solvent control (DMSO).

### 4.11. Inhibition of Biofilm Formation

The strains used were the Gram-negative *E. coli* K-12 AG100 and *E. coli* ATCC 25922 and the Gram-positive *S. aureus* ATCC 25923 and *S. aureus* MRSA 272123. The detection of biofilm formation was possible with the use of the dye crystal violet (CV; 0.1% (*v*/*v*)). The initial inoculum was incubated in LB (for *E. coli*) or in TSB (for *S. aureus*) overnight, and then diluted to an OD_600_ of 0.1. Then, the bacterial suspension was added to 96-well microtiter plates and the extracts and EO were added at half the MIC. The pure compounds were added at 100 µM and lower concentrations. The final volume in each well was 200 µL. CCCP for *E. coli* strains and thioridazine (TZ) for *S. aureus* strains were used as positive controls. The plates were incubated at 30 °C for 48 h, with gentle stirring (100 rpm). After incubation, the TSB medium was discarded, and unattached cells were removed by washing the plates with tap water. Then, 200 µL CV was added to the wells and incubated for 15 min at room temperature (~24 °C). In the next step, CV was eliminated from the wells, and the plates were washed again with tap water, and 200 µL of 70% ethanol was given to the wells. The biofilm formation was established by measuring the OD_600_ using a Multiscan EX ELISA plate reader (Thermo Labsystems, Cheshire, WA, USA). The antibiofilm effect of the samples was expressed in the percentage (%) of decrease of biofilm formation.

## 5. Conclusions

The present study describes the antibacterial evaluation of MeOH, *n*-hexane extracts, EO of *O. majorana* and its monoterpene constituents. It can be concluded that EO has a potent antibacterial activity against *S. aureus*, *S. aureus* MRSA, *S. epidermidis*, *E. coli*, *E. coli* AG-100, and *K. pneumoniae* bacteria when tested in undiluted form. Terpinen-4-ol, α-terpinene, and linalool have the most substantial contribution to the antibacterial effect of EO against *E. coli* and *S. aureus* strains, because these compounds exhibited the highest efficacy (56–61 mM).

In addition to the antibacterial activity, EO exhibited a pronounced resistance-reversing effect against resistant and sensitive *E. coli* strains, and among the EO constituents, sabinene was found to be partly responsible for this activity. On the resistant *S. aureus* MRSA strain, moderate efflux pump inhibitory activity was demonstrated by the monoterpenes containing *n*-hexane extract and EO, with sabinene hydrate to be partly responsible for this effect. 

Moreover, the biofilm formation inhibitory activity of EO on *S. aureus* and *E. coli* AG100, and its monoterpene constituents on *S. aureus* MRSA and *E. coli* were confirmed. The MeOH and *n*-hexane extracts were promising only for the antibiofilm activity. Sabinene-hydrate demonstrated inhibitory effects on both the efflux mechanisms and the biofilm formation; such results might raise the possibility of a common mechanism of action in the background. These results could be the starting points for further investigations aiming to find potential antibacterial components with a higher affinity against resistant bacteria.

## Figures and Tables

**Table 1:** Chemical composition of essential oil of Hungarian variety of *O. majorana*.

	Compounds ^a^	RI ^b^	% in Sample
1	α-thujene	924	0.51
2	α-pinene	932	0.43
3	sabinene	972	4.53
4	β-pinene	978	0.26
5	myrcene	987	1.23
6	α-phelladrene	1007	0.14
7	α-terpinene	1017	3.00
8	*p*-cymene	1024	4.72
9	limonene	1028	0.20
10	β-phelladrene	1030	2.14
11	β-ocimene	1044	0.10
12	γ-terpinene	1057	6.48
13	*cis*-sabinene hydrate	1070	5.44
14	terpinolene	1085	1.62
15	linalool	1101	0.10
16	*trans*-sabinene hydrate	1103	25.18
17	*cis-p*-menth-2-en-1-ol	1125	2.35
18	*trans-p*-menth-2-en-1-ol	1142	1.34
19	terpinen-4-ol	1182	24.92
20	α-terpineol	1196	4.43
21	linalyl acetate	1249	1.92
22	bornyl acetate	1284	0.27
23	terpin-1-en-4-yl acetate	1295	0.36
24	carvacrol	1298	0.27
25	*trans*-geranyl acetate	1377	0.28
26	β-caryophyllene	1417	1.98
27	spatulenol	1574	0.47
		**Total**	**94.67**

^a^ Compounds listed in sequence of elution from a DB-5 MS column. ^b^ Retention indices calculated against C8 to C32 *n*-alkanes on a DB-5MS column.

**Table 2:** Antimicrobial activities of marjoram samples at 50 µg/mL concentration by agar disc diffusion method (diameter of inhibition zone, mm) ^a, b, c^.

Sample	*S. aureus ATCC 29213*	*S. aureus* MRSA ATCC 43300	*S. epidermidis* ATCC 12228	*E. faecalis* ATCC 29212	*E. coli* ATCC 35218	*E. coli* AG-100	*K. pneumoniae* ATCC 700603	*P. aeruginosa* ATCC 27853	*C. albicans* ATCC 10231
MeOH extract	7 ± 0.3	7 ± 0.0	0	0	0	0	0	0	0
*n*-hexane extract	7 ± 0.5	7 ± 0.3	7 ± 0	0	8 ± 0.2	7 ± 0	8 ± 0	7 ± 0.2	7 ± 0
Essential oil	0	0	0	0	7 ± 0.2	0	7 ± 0.1	0	0
Essential oil ^c^	**16** ± 0.5	**14** ± 0.3	**14** ± 0	7 ± 0.3	**14** ± 0.4	**14** ± 0.4	**10** ± 0.3	8 ± 0	**13** ± 0.3
DMSO	0	0	0	0	0	0	0	0	0

^a^ Values are average of three consecutive trial; ^b^ Zone of inhibition in mm including the disc diameter (6 mm); ^c^ Direct application of essential oil (undiluted).

**Table 3:** MIC values of the extracts and essential oil of *O. majorana*.

Samples	*S. aureus* ATCC 25923	*S. aureus* MRSA ATCC 43300	*E. coli* ATCC 25922	*E. coli* AG100
MeOH extract	>100 µg/mL	>100 µg/mL	>100 µg/mL	>100 µg/mL
*n-*hexane extract	>100 µg/mL	>100 µg/mL	>100 µg/mL	>100 µg/mL
Essential oil	0.125%	0.125%	0.125%	0.250%

**Table 4:** Minimum inhibitory concentrations (MICs) of *O. majorana* essential oil components.

Compounds	*S. aureus* ATCC 29213	*S. aureus* MRSA ATCC 43300	*E. coli* ATCC 35218	*E. coli* AG100
linalool	>10 µL/mL >56 mM	>10 µL/mL >56 mM	10 µL/mL 56 mM	10 µL/mL 56 mM
sabinene	10 µL/mL >62 mM	10 µL/mL >62 mM	>10 µL/mL >62 mM	>10 µL/mL >62 mM
sabinene-hydrate *	>0.154 mg/mL >62 mM	>0.154 mg/mL >62 mM	>0.154 mg/mL >62 mM	>0.154 mg/mL >62 mM
α-terpinene	10 µL/mL 61 mM	10 µL/mL 61 mM	10 µL/mL 61 mM	10 µL/mL 61 mM
γ-terpinene	>10 µL/mL >62 mM	>10 µL/mL >62 mM	>10 µL/mL >62 mM	>10 µL/mL >62 mM
terpinen-4-ol	10 µL/mL 60 mM	10 µL/mL 60 mM	5 µL/mL 30 mM	5 µL/mL 30 mM

* Sabinene hydrate is a solid material at room temperature, therefore its concentration is given in mg/mL.

**Table 5:** Relative fluorescence index (RFI) of tested extracts, essential oil and its constituents against *E. coli* ATCC 25922, *E. coli* AG100, *S. aureus* ATCC 25923 and *S. aureus* MRSA ATCC 43300.

Relative Fluorescence Index (RFI) ^1^				
	*E. coli* ATCC 25922	*E. coli* AG100	*S. aureus* ATCC 25923	*S. aureus* MRSA ATCC 43300
	0.0625%	0.125%	0.0625%	0.0625%
MeOH extract	−0.14	−0.17	−0.17	−0.52
*n-*hexane extract	0.04	−0.12	−0.06	**0.39**
Essential oil	**4.27**	**1.27**	0.19	**0.35**
	0.0312%	0.0625%		
MeOH extract	−0.10	−0.18		
*n-*hexane extract	−0.09	−0.13		
Essential oil	**3.21**	**0.64**		
	100 µM	100 µM	100 µM	100 µM
α-terpinene	0.07	−0.00	0.06	0.00
γ-terpinene	0.01	−0.03	0.06	−0.01
terpinen-4-ol	0.07	−0.00	0.02	−0.01
sabinene	**0.25**	−0.02	0.06	0.10
sabinene hydrate	0.02	−0.04	0.00	**0.27**
linalool	−0.01	−0.05	0.03	−0.05
	50 µM	50 µM		
α-terpinene	0.02	0.01		
γ-terpinene	0.05	−0.01		
terpinen-4-ol	0.07	−0.02		
sabinene	0.04	−0.04		
sabinene hydrate	−0.02	−0.05		
linalool	−0.02	−0.08		
CCCP ^2^	**2.00**	**0.70**		
RES ^3^		−	**1.55**	**1.06**

^1^ SD values were calculated and given in Tables S2–S5. ^2^ CCCP carbonyl cyanide 3-chlorophenylhydrazone; ^3^ RES reserpine; the active compounds are presented in bold.

**Table 6:** Antibiofilm effect of extracts, essential oil of *O. majorana* and its monoterpene components on sensitive and resistant *E. coli* strains ^1^.

Sample	Inhibition %			
	*E. coli* ATCC 25922		*E. coli* AG100	
	0.0625%	0.0312%	0.125%	0.0625%
MeOH extract	−5.77 **	55.61 *	−59.60 ^ns^	−56.68 ^ns^
*n-*hexane extract	57.79 *	**64.43 ^ns^**	−13.74 *	−41.58 **
Essential oil	−104.64	**–**	**104.16**	**–**
	100 µM/	50 µM	100 µM	50 µM
α-terpinene	1.95 *	17.68 **	−53.71 ^ns^	−35.98 *
γ-terpinene	34.34 **	**37.80** *	−33.37 ^ns^	−13.62 ^ns^
terpinene 4-ol	−2.09 ***	**42.36** ^ns^	−28.19 ^ns^	8.51 ^ns^
sabinene	36.35 **	**48.57** ^ns^	−59.78 ^ns^	15.40 ^ns^
sabinene hydrate	37.93 ***	**55.97** *	−42.47 ^ns^	12.13 *
linalool	28.98 ***	**49.68** ^ns^	−0.89 *	−11.78 *
CCCP		**63.37**		**50.14**

^1^ The measurements were performed in triplicate. * *p* < 0.05, ** *p* < 0.01, *** *p* < 0.001, ns: not significant.

**Table 7:** Antibiofilm effect of extracts, essential oil of *O. majorana* and its monoterpene components on sensitive and resistant *S. aureus* strains ^1^.

Sample	Inhibition %			
	*S. aureus* ATCC 25923		*S. aureus* MRSA ATCC 43300	
	0.0625%	0.0312%	0.0625%	0.0312%
MeOH extract	0.93 **	−1.93 **	44.99 **	39.69 *
*n-*hexane extract	1.22 **	0.46 **	−53.03 ^ns^	18.37 **
Essential oil	**69.24**	–	4.38	–
	100 µM	50 µM	100 µM	50 µM
α-terpinene	1.47 **	−1.13 **	−64.81 ^ns^	26.82 **
γ-terpinene	0.76 **	−1.91 ***	−125.22 *	−53.03 *
terpinene 4-ol	−0.64 **	−1.51 **	66.31 **	53.71 **
sabinene	0.61 **	−1.83 ***	53.53 **	86.26 *
sabinene hydrate	−0.80 **	−2.84 **	60.56 **	69.48 **
linalool	0.21 **	−2.58 **	34.44 **	28.87 *
DMSO	0.56		−64.81	
TZ	−	**97.07**		**94.66**

^1^ The measurements were performed in triplicate. * *p* < 0.05, ** *p* < 0.01, *** *p* < 0.001, ns: not significant.

## Data Availability

Not applicable.

## Supplementary Materials

The following supporting information can be downloaded at: https://www.mdpi.com/article/10.3390/plants11111432/s1: Figure S1: GC chromatogram of *n*-hexane extract of *Origanum majorana*; Table S1: Composition of the *n*-hexane extract of *Origanum majorana* determined by GC-MS; Table S2–S4. Efflux pump inhibitory activity of marjoram extracts, essential oil, and its constituents by real time ethidium bromide accumulation assay on *E. coli* AG100, *E. coli* ATCC 25922, *S. aureus* ATCC 25923, and *S. aureus* MRSA ATCC 43300 strains.
