# Supplementary material for: Antimicrobial, Multidrug Resistance Reversal and Biofilm Formation Inhibitory Effect of Origanum majorana Extracts, Essential Oil and Monoterpenes
Source: Plants (Basel). 2022 May 27;11(11):1432. doi: 10.3390/plants11111432 (PMC9183178; doi:10.3390/plants11111432)
Supplement: Supplementary file 1 [file plants-11-01432-s001.zip › plants-1695266-supplementary.pdf]

SUPPORTING MATERIAL

Antimicrobial, multidrug resistance reversal and biofilm formation inhibitory effect of *Origanum majorana* extracts, essential oil and monoterpenes

Tasneem Sultan Abu Ghazal,[1] Zsuzsanna Schelz,[2] Lívia Vidács,[1] Nikoletta Szemerédi,[3] Katalin Veres,[1] Gabriella Spengler,[3] Judit Hohmann[1,4*]

Department of Pharmacognosy, University of Szeged, 6720 Szeged, Hungary
Department of Pharmacodynamics and Biopharmacy, University of Szeged, 6720 Szeged, Hungary
Department of Medical Microbiology, Albert Szent-Györgyi Health Center and Albert Szent-Györgyi Medical School, University of Szeged, Semmelweis utca 6, H-6725 Szeged, Hungary
Interdisciplinary Centre for Natural Products, University of Szeged, Eötvös u. 6, H-6720 Szeged, Hungary

CONTENT

Figure S1. GC chromatogram of *n*-hexane extract of *Origanum majorana*…………………..2

Table S1. Composition of the *n*-hexane extract of *Origanum majorana* determined by GC-MS…………………………………………………………………………2

Table S2. Efflux pump inhibitory activity of marjoram extracts, essential oil and its constituents by real time ethidium bromide accumulation assay on *E. coli* AG100 strain …………………4

Table S3. Efflux pump inhibitory activity of marjoram extracts, essential oil and its constituents by real time ethidium bromide accumulation assay on *E. coli* ATCC 25922 strain ……………………………………………………………………5

Table S4. Efflux pump inhibitory activity of marjoram extracts, essential oil and its constituents by real time ethidium bromide accumulation assay on *S. aureus* ATCC 25923 strain ………..6

Table S5. Efflux pump inhibitory activity of marjoram extracts, essential oil and its constituents by real time ethidium bromide accumulation assay on *S. aureus* MRSA ATCC 43300 strain ……………………………………………………………………7

Figure S1. GC chromatogram of *n*-hexane extract of *Origanum majorana*

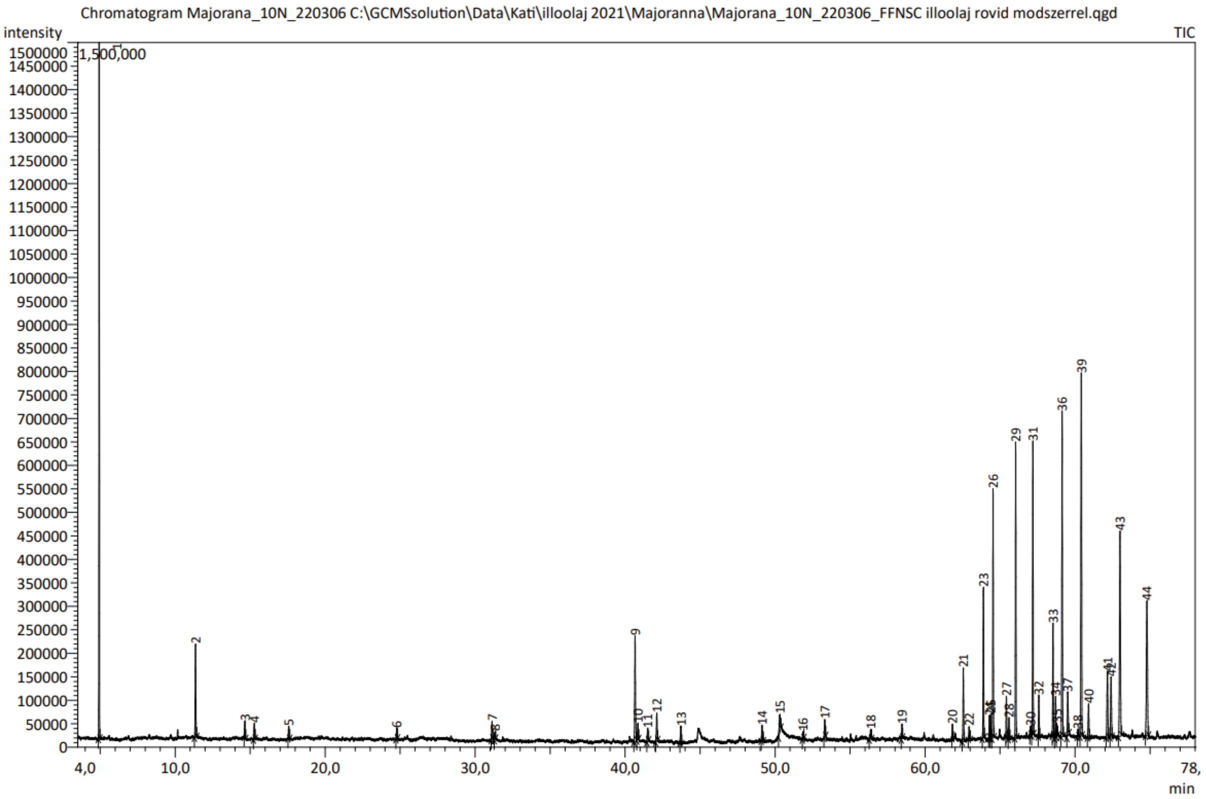

Table S1. Composition of the *n*-hexane extract of *Origanum majorana* determined by GC-MS

Peak	Name	RI	RT (min)	Area
1	*n*-nonane	896	4.935	1043259
2	***trans*-sabinene hydrate**	1099	11.357	76535
3	**terpinen-4-ol**	1177	14.65	22450
4	**terpineol <alpha->**	1192	15.277	19055
5	**linalyl acetate**	1245	17.574	19545
6	**β-caryophyllene**	1413	24.784	7847
7	**spathulenol**	1570	31.134	9087
8	caryophyllene oxide	1574	31.327	tr
9	neophytadiene	1829	40.669	63230
10	phytone	1835	40.865	17650
11	neophytadiene	1854	41.516	9549
12	heptadec-(4*E*)-enol	1872	42.112	15635
13	unknown	1920	43.724	15064
14	methyl-linolenate	2089	49.125	12930

15	ethyl-linolenate	2131	50.329	tr
16	unknown	2185	51.862	tr
17	unknown	2243	53.306	32002
18	unknown	2381	56.371	15395
19	*n*-pentacosane	2487	58.455	15264
20	unknown	2652	61.821	15431
21	*n*-heptacosane	2688	62.552	83059
22	unknown	2709	62.939	34814
23	unknown	2761	63.891	165279
24	unknown	2783	64.288	21987
25	*n*-octacosane	2788	64.369	24549
26	squalene	2797	64.535	346114
27	unknown	2850	65.424	44384
28	unknown	2860	65.594	19484
29	*n*-nonacosane	2887	66.041	305629
30	unknown	2949	67.028	9378
31	*n*-pentacosane	2959	67.191	292679
32	*n*-triacontane	2984	67.582	48095
33	unknown	3041	68.528	115274
34	*n*-pentatriacontane	3052	68.703	40438
35	unknown	3056	68.783	6034
36	unknown	3078	69.137	363428
37	unknown	3100	69.513	91178
38	*n*-hexatriacontane	3141	70.204	9182
39	unknown	3154	70.414	489964
40	*n*-dotriacontane	3183	70.911	48160
41	*n*-octatriacontane	3258	72.161	100530
42	*n*-octatriacontane	3272	72.4	91952
43	*n*-tritriacontane	3308	72.996	318101
44	*n*-tetratriacontane	3415	74.789	244192

Table S2. Efflux pump inhibitory activity of marjoram extracts, essential oil and its constituents by real time ethidium bromide accumulation assay on *E. coli* AG100 strain

***E. coli* AG100**	**Mean**	**SD**	**RFI**
**Extracts**	**125 µg/mL**		
MeOH extract	19310	98.97474	-0.1651
n-hexane extract	20402	1749.647	-0.1179
Essential oil	52568	1322.622	**1.2729**
	**62.5 µg/mL**		
MeOH extract	19045	103.1843	-0.1766
n-hexane extract	20118	121.3768	-0.1301
Essential oil	37865	1056.015	**0.6372**
**Compounds**	**100 µM**		
α-terpinene	23049.33	273.7158	-0.0034
γ-terpinene	22380.67	315.3321	-0.0323
terpinene-4-ol	23107	478.091	-0.0009
sabinene	22778.67	603.6094	-0.0151
sabinene hydrate	22168	53.11309	-0.0415
linalool	22026.33	419.6931	-0.0476
	**50 µM**		
α-terpinene	23265.67	1054.827	0.0059
γ-terpinene	22887	551.1361	-0.0104
terpinene-4-ol	22729	676.5006	-0.0173
sabinene	22180	285.454	-0.0410
sabinene hydrate	21983.67	1085.728	-0.0495
linalool	21179.67	122.4105	-0.0843
**Controls**			
DMSO	23128	2065.409	
E. coli AG100	21553.33	637.2891	-0.0681
CCCP	39281.67	156.5162	**0.6985**

Table S3. Efflux pump inhibitory activity of marjoram extracts, essential oil and its constituents by real time ethidium bromide accumulation assay on *E. coli* ATCC 25922 strain

***E. coli* ATCC 25922**	**Mean**	**SD**	**RFI**
**Extracts**	**62.5 µg/mL**		
MeOH extract	22441.33	1094.717	-0.1384
n-hexane extract	27121	1888.161	0.0412
Essential oil	137226.7	1508.068	**4.2684**
	**31.2 µg/mL**		
MeOH extract	23364.67	544.6332	-0.1030
n-hexane extract	25818	1202.07	-0.0088
Essential oil	109732.3	7022.362	**3.2129**
**Compounds**	**100 µM**		
α-terpinene	27843.33	877.377	0.0690
γ-terpinene	26368.33	350.3574	0.0123
terpinene-4-ol	27878.67	883.148	0.0703
sabinene	32590.67	10117.96	0.2512
sabinene hydrate	26600	631.3153	0.0212
linalool	25667.67	344.6452	-0.0146
	**50 µM**		
α-terpinene	26550	107.5314	0.0193
γ-terpinene	27434.67	82.97188	0.0533
terpinene-4-ol	27748	692.242	0.0653
sabinene	27145.67	1438.72	0.0422
sabinene hydrate	25548.67	835.0032	-0.0191
linalool	25402.33	344.1138	-0.0248
**Controls**			
DMSO	26047	1128.075	
E. coli ATCC	26708.67	316.0575	0.0254
CCCP	78192.33	475.7534	**2.0020**

Table S4. Efflux pump inhibitory activity of marjoram extracts, essential oil and its constituents by real time ethidium bromide accumulation assay on *S. aureus* ATCC 25923 strain

S. aureus **ATCC 25923**	**Mean**	**SD**	**RFI**
**Extracts**	**62.5 µg/mL**		
MeOH extract	19704.67	1398.57	-0.17
n-hexane extract	22409.33	1124.71	-0.06
Essential oil	21324.00	954.24	**0.19**
**Compounds**	**100 µM**		
α-terpinene	25220.00	638.77	0.06
γ-terpinene	25275.67	1974.12	0.06
terpinene-4-ol	24275.33	573.93	0.02
sabinene	25065.33	570.63	0.06
sabinene hydrate	23667.67	1569.13	0.00
linalool	24364.00	1147.47	0.03
**Controls**			
DMSO	23751.00	389.83	
S. aureus ATCC	26062.00	412.66	0.10
RES	60508.67	5406.55	**1.55**

Table S5. Efflux pump inhibitory activity of marjoram extracts, essential oil and its constituents by real time ethidium bromide accumulation assay on *S. aureus* MRSA ATCC 43300 strain

***S. aureus* MRSA ATCC 43300**	**Mean**	**SD**	**RFI**
**Extracts**	**62.5 µg/mL**		
MeOH extract	32845.33	1461.43	-0.52
n-hexane extract	95286.33	4736.54	**0.39**
Essential oil	65466.80	779.93	**0.35**
**Compounds**	**100 µM**		
α-terpinene	68390.67	1351.32	0.00
γ-terpinene	67851.33	741.31	-0.01
terpinene-4-ol	67717.33	1342.24	-0.01
sabinene	74905.00	2669.34	0.10
sabinene hydrate	87137.33	29123.07	**0.27**
linalool	65269.33	3227.53	-0.05
**Controls**			
DMSO	68370.33	894.34	
S. aureus MRSA 43300	72053.67	3033.87	0.05
RES	140833.33	103221.54	**1.06**
